# Expression, crystal structure and cellulase activity of the thermostable cellobiohydrolase Cel7A from the fungus *Humicola grisea* var. *thermoidea*

**DOI:** 10.1107/S1399004714013844

**Published:** 2014-08-29

**Authors:** Majid Haddad Momeni, Frits Goedegebuur, Henrik Hansson, Saeid Karkehabadi, Glareh Askarieh, Colin Mitchinson, Edmundo A. Larenas, Jerry Ståhlberg, Mats Sandgren

**Affiliations:** a Department of Chemistry and Biotechnology, Swedish University of Agricultural Sciences, PO Box 7015, SE-750 07 Uppsala, Sweden; b DuPont, Industrial Biosciences, Archimedesweg 30, 2333 CN Leiden, The Netherlands; c DuPont, Industrial Biosciences, Page Mill Road, Palo Alto, CA 94304, USA

**Keywords:** cellobiohydrolase, Cel7A

## Abstract

Glycoside hydrolase family 7 (GH7) cellobiohydrolases (CBHs) play a key role in biomass recycling in nature. They are typically the most abundant enzymes expressed by potent cellulolytic fungi, and are also responsible for the majority of hydrolytic potential in enzyme cocktails for industrial processing of plant biomass. The thermostability of the enzyme is an important parameter for industrial utilization. In this study, Cel7 enzymes from different fungi were expressed in a fungal host and assayed for thermostability, including *Hypocrea jecorina* Cel7A as a reference. The most stable of the homologues, *Humicola grisea* var. *thermoidea* Cel7A, exhibits a 10°C higher melting temperature (*T*_m_ of 72.5°C) and showed a 4–5 times higher initial hydrolysis rate than *H. jecorina* Cel7A on phosphoric acid-swollen cellulose and showed the best performance of the tested enzymes on pretreated corn stover at elevated temperature (65°C, 24 h). The enzyme shares 57% sequence identity with *H. jecorina* Cel7A and consists of a GH7 catalytic module connected by a linker to a C-terminal CBM1 carbohydrate-binding module. The crystal structure of the *H. grisea* var. *thermoidea* Cel7A catalytic module (1.8 Å resolution; *R*_work_ and *R*_free_ of 0.16 and 0.21, respectively) is similar to those of other GH7 CBHs. The deviations of several loops along the cellulose-binding path between the two molecules in the asymmetric unit indicate higher flexibility than in the less thermostable *H. jecorina* Cel7A.

## Abstract 2

Cellobiohydrolase Cel7A from *H. grisea* var. *thermoidea* showed a 10°C higher *T*_m_ and a 75% higher yield than *H. jecorina* Cel7A in a performance assay at 65°C. The crystal structure at 1.8 Å resolution indicates higher flexibility in tunnel-defining loops and reveals a new loop conformation near the active centre.

## 1. Introduction   

The global carbon cycle is fundamentally dependent on the digestion of cellulosic biomass (Malhi, 2002 ▶). Cellulose is the main component of plant cell walls and is one of the most abundant natural resources available for the production of renewable energy. It is a linear polymer composed of β-1,4-linked d-glucose units. In nature, cellulose is degraded by microorganisms through the synergistic action of hydrolytic enzymes commonly assigned as cellulases. Three distinct classes of cellulases have been recognized: endoglucanases (EGs; EC 3.2.1.4), cellobiohydrolases (CBHs; EC 3.2.1.91 and 3.2.1.176) and β-glucosidases (Bgls; EC 3.2.1.21). EGs hydrolyse cellulose chains internally, whereas CBHs cleave off cellobiose units from either the reducing or the nonreducing end of the cellulose polymer (Schmid & Wandrey, 1990 ▶; Vršanská & Biely, 1992 ▶; Divne *et al.*, 1998 ▶; Ståhlberg *et al.*, 1996 ▶). Lastly, β-glucosidases are able to complete the degradation process by hydrolysing soluble oligosaccharides to glucose (Gilkes *et al.*, 1991 ▶; Lynd *et al.*, 2002 ▶).

Cellulases, both CBHs and EGs, typically comprise a modular architecture. A common fungal cellulase architecture contains a catalytic domain (CD) and a smaller carbohydrate-binding module (CBM) connected *via* a highly glycosylated linker (Tomme *et al.*, 1988 ▶; van Tilbeurgh *et al.*, 1986 ▶). Cellulases are glycoside hydrolases, which have been grouped into families and clans in the Carbohydrate Active enZYmes (CAZY) database based on similarities in sequence, structure and enzymatic mechanism (Henrissat & Bairoch, 1996 ▶; Henrissat & Davies, 1997 ▶).

Glycoside hydrolase family 7 (GH7) CBHs have been identified as the major protein secreted under cellulase-inducing conditions in several different fungi (Nummi *et al.*, 1983 ▶;Muñoz *et al.*, 2001 ▶; Momeni *et al.*, 2013 ▶) and play a key role in the degradation of plant biomass, both industrially and in nature. They act processively from the reducing end of a cellulose chain (Davies & Henrissat, 1995 ▶; Boisset *et al.*, 2000 ▶; Kipper *et al.*, 2005 ▶). Three-dimensional structures of eight GH7 CBHs have been reported previously. *Hypocrea jecorina* Cel7A (*Hje*Cel7A; Divne *et al.*, 1994 ▶), *Trichoderma harzianum* Cel7A (*Tha*Cel7A; Textor *et al.*, 2013 ▶), *Phanerochaete chrysosporium* Cel7D (*Pch*Cel7D; Muñoz *et al.*, 2001 ▶) and *Heterobasidion irregulare* Cel7A (*Hir*Cel7A; Momeni *et al.*, 2013 ▶) are secreted by mesophilic fungi, whereas *Melanocarpus albomyces* Cel7B (*Mal*Cel7B; Parkkinen *et al.*, 2008 ▶) and *Rasamsonia emersonii* (formerly *Talaromyces emersonii*) Cel7A (*Rem*Cel7A; Grassick *et al.*, 2004 ▶) are from thermophilic fungi. Recently, the structure of the CBH Cel7B from the marine wood borer *Limnoria quadripunctata* (*Lqu*Cel7B) has been determined (Kern *et al.*, 2013 ▶).

The most significant structural feature of GH7 CBHs is the presence of a 50 Å long cellulose-binding tunnel in which up to 11 subsites for binding of glucose residues from a cellulose chain have been identified (Divne *et al.*, 1998 ▶). These subsites are numbered −7 to +4 from the nonreducing end to the reducing end of the cellulose chain, with the catalytic centre located between subsites −1 and +1 (Biely *et al.*, 1981 ▶; Davies *et al.*, 1997 ▶). Four highly conserved tryptophan residues form sugar-binding platforms at subsites −7, −4, −2 and +1. GH7 CBHs exhibit high sequence identity (>50%) and the fold and active site are highly conserved. Variations, presumably related to function, occur primarily in the length and sequence of the loops that build up the substrate-binding tunnel. One such loop, the so-called exo-loop, of *Hje*Cel7A has been shown to contribute to a higher degree of processivity compared with that of *Pch*Cel7D (von Ossowski *et al.*, 2003 ▶). The dynamic behaviour of loop regions differs significantly between these enzymes in molecular-dynamics (MD) simulations, which probably relates to differences in processivity, endo-initiation and product inhibition (Momeni *et al.*, 2013 ▶).

The economics of the industrial-scale enzymatic conversion of biomass to fermentable sugars would benefit from improved thermostability of the enzyme mixtures used (Viikari *et al.*, 2007 ▶) since the lifetime of the cellulases are expected to increase with thermostability. Thus, thermostable cellulases are good candidates for use in industrial biomass-conversion processes since higher thermal stability could lead to higher specific activity at elevated temperatures and to a shorter hydrolysis time.

The thermophilic fungus *Humicola grisea* var. *thermoidea* has been shown to produce several different CBHs and EGs with pronounced activity at elevated temperatures (Takashima *et al.*, 1996 ▶). The three-dimensional structure of only one enzyme from *H. grisea*, the EG Cel12A, has been reported (Sandgren *et al.*, 2004 ▶).

In this study, we report the crystallization, structural determination and biochemical characterization of *H. grisea* var. *thermoidea* Cel7A (*Hgt*Cel7A). The results are discussed in the light of differences and similarities compared with other mesophilic and thermophilic GH7 cellobiohydrolases.

## 2. Materials and methods   

### 2.1. Cloning of Cel7A-encoding genes   

Fungal strains were grown on potato dextrose agar plates and genomic DNA was isolated using the FastPrep method according to the manufacturer’s instructions (Qbiogene Inc., Carlsbad, California, USA). The system consists of the FastPrep instrument as well as FastPrep kits for nucleic acid isolation.

Primers for *Hje*Cel7A were used to amplify homologous sequences in genomic DNA isolated from a subset of *Hypocrea* strains kindly provided by Professor Dr C. P. Kubicek, including *H. orientalis*, *H. schweinitzii*, *Trichoderma pseudokoningii* and *T. konilangbra*. Gene-specific primers for the *T. citrinoviride* Cel7 were made after receiving sequence information from Professor Dr C. P. Kubicek, while primers for the other strains were developed from published sequences as indicated in Table 1 ▶. For *H. grisea* var. *thermoidea*, homologous 5′ (PVS203) and 3′ (PVS204) primers were based on the sequence of Cel7A from *H. grisea* var. *thermoidea* (IFO9854 sequence D63515). The sequence of PVS203 without *attB*1 was 5′-ATGCGTACCGCCAAGTTCGC-3′ and the sequence of PVS204 without *attB*2 was 5′-TTACAGGCACTGAGAGTACCAG-3′.

PCR was performed using 20 µl 5× reaction buffer comprising 50 m*M* Tris–HCl pH 8.5, 87.5 m*M* ammonium sulfate, 6.25 m*M* MgCl_2_, 2.5%(*v*/*v*) Tween 20, 7.5%(*v*/*v*) DMSO, 0.2 m*M* each of dATP, dTTP, dGTP and dCTP, 1 µl 100 ng µl^−1^ genomic DNA, 1 µl Tgo Polymerase (Roche Diagnostics GmbH, catalogue No. 3186199) at one unit per microlitre, 0.2 µ*M* of each primer and water to 100 µl. The PCR reaction was performed on a PTC-200 Peltier Thermal Cycler (MJ Research Inc.) under the following conditions with *H. grisea* var. *thermoidea* and other homologous primer/template amplifications: one cycle of 1 min at 96°C followed by 30 cycles of 30 s at 94°C, 60 s at 55°C, 2 min at 72°C and one cycle of 7 min at 72°C; the temperature was then lowered to 15°C for storage and further analysis. For the heterologous amplifications using *Hje*Cel7A primers and closely related templates, the annealing temperature was lowered to 45°C and was ramped to 55°C in ten cycles.

Each Cel7 PCR fragment was cloned into plasmid pDONR201 ([Km^r^]; Invitrogen) and transformed into *Escherichia coli* strain MAX Efficiency DH5α ([ϕ80d*lac*ZΔM15 Δ(*lac*ZYA-*arg*F) U169 deoR *rec*A1 *end*A1 *hsd*R17(r_k_^−^, m_k_^+^) *pho*A *sup*E44 λ^−^ thi-1 *gyr*A96 *rel*A1]; Invitrogen). General recombinant DNA procedures were adapted from Sambrook & Gething (1989 ▶). The cloned Cel7 genes were sequenced by BaseClear (Holding BV, Leiden, The Netherlands) and were analysed using the *VectorNTI* software package. The Cel7 genes were transferred to *Aspergillus niger* var. *awamori* AP4 for expression as described below, and in the case of *H. grisea* var. *thermoidea* also into *H. jecorina*.

### 2.2. Protein expression and purification   

Each Cel7 DNA construct was transferred to the *E. coli*/*A. niger* shuttle expression vector pRAXdes (Goedegebuur *et al.*, 2013 ▶), where the target gene is expressed under the control of the glucoamylase promoter from *A. nidulans*. Each Cel7 gene carried its native signal sequence from the original host.

The *E. coli* transformants were isolated from ampicillin agar plates and plasmid DNA isolation was performed. Plasmids carrying the Cel7-coding gene were then transformed into *A. niger* var. *awamori* AP4 (Berka & Barnett, 1989 ▶) according to the method described by Cao *et al.* (2000 ▶). Spores of the *A. niger* var. *awamori* transformants were germinated and grown in minimal medium lacking uridine (Ballance *et al.*, 1983 ▶). Spores from a single colony were spread on a fresh minimal medium with sorbitol (MMS) plate and left for sporulation. The enzymes were produced by inoculating 500 ml baffled shake flasks with spore suspension from 1 cm^2^ of sporulating fungal mycelium and cultivation for 3 d at 37°C as described by Cao *et al.* (2000 ▶).

The Cel7 enzymes were purified by hydrophobic interaction chromatography on Bio-Rad Poly-Prep columns packed with 1 ml Phenyl Sepharose (GE Healthcare) and equilibrated with five column volumes (CV) of buffer *A* (0.5 *M* ammonium sulfate, 20 m*M* sodium phosphate pH 6.8). Ammonium sulfate (4 *M*) was added to the culture filtrate to 0.5 *M* concentration and 2 CV were applied to the column followed by washing with 5 CV buffer *A*. The Cel7 enzyme was then eluted with 4 CV 20 m*M* sodium phosphate pH 6.8.

In another procedure, the Cel7A gene from *H. grisea* var. *thermoidea* was inserted into the *E. coli*/*H. jecorina* shuttle vector pTREX2g (Baldwin *et al.*, 2008 ▶), where the gene is expressed under the control of the *cbh1* promoter from *H. jecorina*, containing the *amdS* (acetamidase) selection marker. The plasmid was transformed into a strain of *H. jecorina* deleted for *cbh1* ^−^, *cbh2* ^−^, *egl1* ^−^, *egl2* ^−^ as described by Bower *et al.* (1998 ▶). Spores of *H. jecorina* transformants were propagated on defined-medium agar plates containing acetamide as the nitrogen source (Penttilä *et al.*, 1987 ▶). Cultivation and enzyme production was performed as described previously (Foreman *et al.*, 2003 ▶).

### 2.3. *T*_m_ measurements   

Protein melting points (*T*_m_) were determined according to the methods of Luo *et al.* (1995 ▶) and Gloss & Matthews (1997 ▶). Circular-dichroism (CD) spectra were collected on an Aviv 215 CD spectrophotometer (Aviv Biomedical Inc., Lakewood, USA) between 210 and 260 nm at 25°C. The buffer conditions were 50 m*M* bis-tris propane, 50 m*M* ammonium acetate/glacial acetic acid at pH 5.5. The protein concentration was kept between 0.25 and 0.5 mg ml^−1^. After determining the optimal wavelength to monitor unfolding, the samples were thermally denatured by ramping the temperature from 25 to 75°C under the same buffer conditions. Data were collected for 5 s every 2°. Partially reversible unfolding was monitored at 230 nm in a 0.1 cm path-length cell.

### 2.4. Activity assays   

Cel7 expression was monitored by measuring activity against 4-methylumbelliferyl-β-d-lactoside (MU-Lac; Sigma Chemicals, catalogue No. M2405), since Cel7s typically show higher activity against fluorogenic and chromogenic lactoside substrates than the corresponding cellobioside substrates (Becker *et al.*, 2001 ▶). 10 µl culture supernatant was mixed with 170 µl 50 m*M* sodium acetate buffer pH 4.5 in a 96-well microtitre plate, followed by the addition of 20 µl 1 m*M* MU-Lac. The initial rate of fluorescence increase was measured at λ_ex_ = 365 nm and λ_em_ = 445 nm at 50°C for 15 min in a Fluostar Galaxy microtitre plate reader (BMG LABTECH, Offenburg, Germany).

Activity on insoluble cellulosic substrates, phosphoric acid-swollen cellulose (PASC) and pretreated corn stover (PCS) was measured as described by Goedegebuur *et al.* (2013 ▶) and is summarized as follows. The substrate was incubated with enzymes in sealed microtitre plates in 50 m*M* sodium acetate pH 5.0 at specified temperatures and with 700 rev min^−1^ agitation. The reaction was terminated by the addition of 100 m*M* glycine buffer pH 11 to reach a final pH of above 10. An aliquot was immediately withdrawn and filtered through a 0.2 µm membrane to remove solids. The amounts of released soluble sugars were quantified by HPLC as described by Baker *et al.* (1998 ▶).

PASC is an amorphous cellulose substrate and was prepared from Avicel as described by Walseth (1952 ▶) and Wood (1971 ▶). The activity of *Hgt*Cel7A and of *Hje*Cel7A on PASC was monitored for 120 min at 38 and 65°C using 6.3 g PASC substrate per litre and 1.6 mg Cel7 enzyme per gram of cellulose. Corn stover consists of the stalks and leaves of the maize plant that remain after the harvesting of corn and is an abundant agricultural residue of industrial relevance. The corn stover was prepared and pretreated with 2%(*w*/*w*) H_2_SO_4_ as described by Schell *et al.* (2003 ▶). The pretreated corn stover (PCS) was used as substrate in a cellulose-conversion activity assay with the Cel7A homologues from *T. pseudokoningii*, *A. niger*, *H. schweinitzii*, *H. jecorina* and *H. grisea* var. *thermoidea*. This assay combines the Cel7 sample to be tested with proteins from the growth of a *H. jecorina cbh1*-deletion strain (*i.e.* lacking native Cel7A owing to disruption of the *cbh1* gene) in about a 1:1 mass ratio. The reaction mixtures, containing 12.7%(*w*/*v*) PCS [approximately 7%(*w*/*v*) cellulose] and a total enzyme dose of 15.5 mg protein per gram of cellulose, were incubated for 24 h at 65°C prior to analysis of soluble sugars by HPLC.

### 2.5. Crystallization, structure determination and model refinement   

Prior to crystallization, the C-terminal linker–CBM1 was removed from the full-length *Hgt*Cel7A enzyme (obtained from the expression in *H. jecorina*) by partial proteolysis with papain, using the same procedure as described for *Hje*Cel7A (Ståhlberg *et al.*, 1996 ▶). Crystals of the catalytic domain for data collection were obtained at 20°C by mixing equal volumes of protein solution (16 mg ml^−1^ protein in 20 m*M* Tris–HCl pH 7.0) and precipitant solution [22%(*w*/*v*) PEG 8000, 0.2 *M* ammonium sulfate] and equilibration against the precipitant solution using the hanging-drop vapour-diffusion technique (McPherson, 1982 ▶). Crystals were briefly immersed in cryoprotectant (25% glycerol in precipitant solution) and immediately flash-cooled and stored in liquid nitrogen until data collection. No ligand was added to the crystal used. A complete single-wavelength X-ray diffraction data set was collected on beamline ID14-1 at the European Synchrotron Radiation Facility (ESRF), Grenoble, France. The diffraction data were indexed and integrated with *MOSFLM* (Leslie & Powell, 2007 ▶) and scaled with *SCALA* in the *CCP*4 program package (Winn *et al.*, 2011 ▶).

The structure of the *Hgt*Cel7A catalytic domain was solved by molecular replacement with *AMoRe* in the *CCP*4 package using a structure of *Hje*Cel7A as the search model (PDB entry 1cel; Divne *et al.*, 1994 ▶). The initial phases were improved by rigid-body refinement in *REFMAC*5 (Murshudov *et al.*, 2011 ▶). Further model building and refinement, including water molecules, was performed by alternating cycles of restrained refinement with *REFMAC*5 and manual inspection and structure adjustments in *Coot* (Emsley & Cowtan, 2004 ▶) against σ_A_-weighted 2*F*_o_ − *F*_c_ and *F*_o_ − *F*_c_ electron-density maps until no further improvement in *R*_work_ and *R*_free_ could be obtained. Statistics of data processing and structure refinement are summarized in Table 2 ▶. Interpretation, structure comparison and preparation of figures were performed using *PyMOL* (DeLano, 2004 ▶). Atomic coordinates and structure factors have been deposited in the PDB with accession code 4csi.

## 3. Results   

### 3.1. Expression of fungal GH7 cellobiohydrolases   

A host/vector system was developed for heterologous expression in the filamentous fungus *A. niger* var. *awamori* AP4. Gene-specific primers were then used against genomic DNA isolated from a diverse set of fungi to amplify GH7 CBH-encoding genes for expression in this system. Ten cloned Cel7 genes, including *H. jecorina* Cel7A (*Hje*Cel7A) as a reference, were successfully expressed (Table 1 ▶), as shown by activity on methylumbelliferyl-β-d-lactoside (MU-Lac) and SDS–PAGE analysis of the culture broth (data not shown). The homologues share 56–97% protein-sequence identity with *Hje*Cel7A. The enzymes from *H. orientalis*, *H. schweinitzii*, *T. pseudokoningii* and *T. konilangbra* are new Cel7 homologues for which sequences have not been published previously. They were obtained from a subset of closely related *Hypocrea* strains (kindly provided by Professor Dr C. P. Kubicek) using primers for *Hje*Cel7A.

The genes were expressed under the control of a constitutive promoter in order to minimize the background of host proteins and potential interference from other carbohydrases. Consequently, the Cel7 enzymes from shake-flask cultivations could be purified to apparent homogeneity in a single hydrophobic interaction chromatography step.

### 3.2. Expression of *Hgt*Cel7A in *H. jecorina*   

*H. grisea* var. *thermoidea* Cel7A (*Hgt*Cel7A) was further expressed under the control of the *cbh1* (Cel7A) promoter in an engineered *H. jecorina* strain that is devoid of production of the four major native cellulases Cel5A, Cel6A, Cel7A and Cel7B. As demonstrated by SDS–PAGE analysis, *Hgt*Cel7A is the most abundantly expressed protein in the culture filtrate (gel shown in Supplementary Fig. S1^1^).

### 3.3. Thermal stability   

Thermostability was assessed by monitoring the thermal denaturation of the proteins by CD spectroscopy and determination of the protein melting temperature (*T*_m_). Table 1 ▶ shows the *T*_m_ values for the expressed Cel7 homologues. Only one of the enzymes, *Hgt*Cel7A, is considerably more thermostable than *Hje*Cel7A, with a 10°C higher melting temperature (*T*_m_ = 72.5°C).

### 3.4. Activity on phosphoric acid-swollen cellulose and pretreated corn stover   

**Figure 1:**
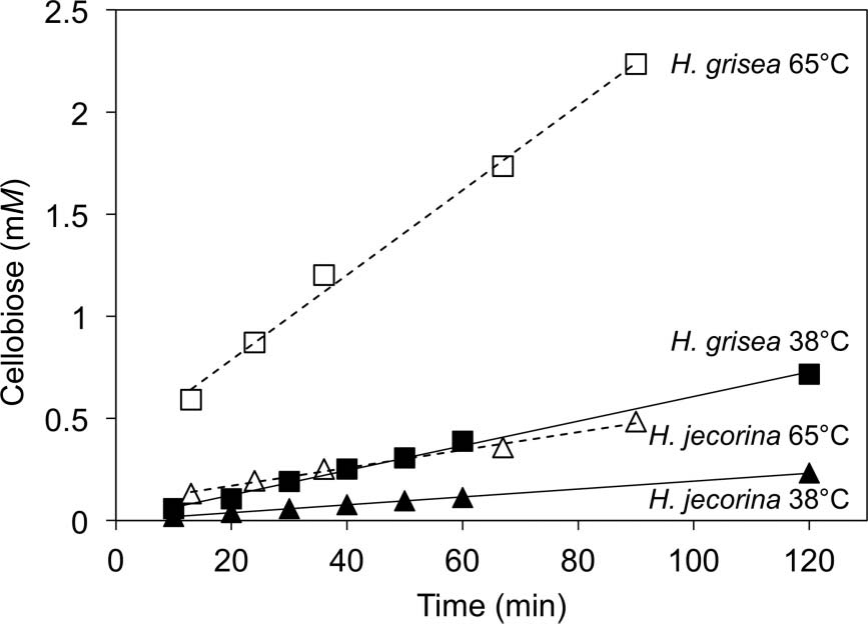
Hydrolysis of phosphoric acid-swollen cellulose (PASC) is faster with *H. grisea* var. *thermoidea* Cel7A than with *H. jecorina* Cel7A at both 38 and 65°C. The reactions contained 6.3 g of PASC per litre in 50 m*M* sodium acetate pH 5.0 and 10 mg of purified *A. niger*-expressed Cel7 enzyme per litre. Soluble sugars were quantified by HLPC.

Comparison of the activity of *Hgt*Cel7A and *Hje*Cel7A when acting alone on phosphoric acid-swollen cellulose (PASC) reveals a much higher hydrolytic rate for *Hgt*Cel7A at both high (65°C; ∼4.8-fold higher initial rate) and moderate (38°C; ∼3.3-fold higher) temperature, as shown in Fig. 1 ▶.

**Figure 2:**
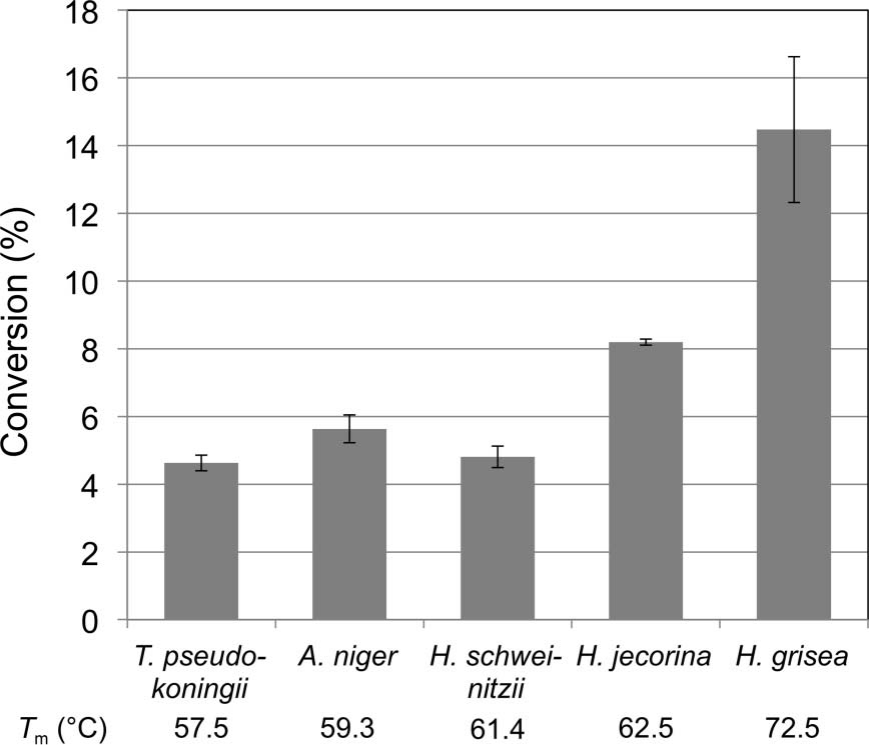
Conversion of pretreated corn stover (PCS) to soluble sugar at 65°C for 24 h by a 1:1 mass ratio of expressed Cel7 and a Cel7A-free *H. jecorina* enzyme cocktail. The reactions contained 12.7% PCS in 50 m*M* sodium acetate pH 5.0 and a total enzyme dose of 15.5 mg protein per gram of cellulose. Soluble sugars were quantified by HPLC.

Cellulosic conversion performance on an industrially relevant lignocellulose biomass material, pretreated corn stover (PCS), was assayed at elevated temperature (65°C for 24 h) for the Cel7s from *T. pseudokoningii*, *A. niger*, *H. schweinitzii*, *H. jecorina* and *H. grisea* var. *thermoidea*. The performance is tested by adding back each Cel7 homologue to the Cel7A-free enzyme cocktail from an engineered *H. jecorina* strain where the *cbh1* gene has been disrupted. As shown in Fig. 2 ▶, the performance on PCS at 65°C correlates with the *T*_m_ values of the Cel7 enzymes, and the highest cellulose conversion was indeed obtained with *Hgt*Cel7A. A 75% higher yield of soluble sugar clearly demonstrates that *Hgt*Cel7A performs better than *Hje*Cel7A at high temperature.

### 3.5. Crystallization, structure solution and quality of the *Hgt*Cel7A structure model   

The C-terminal linker–CBM1 part was proteolytically removed from the full-length *Hgt*Cel7A with papain and the isolated catalytic domain was crystallized, yielding crystals belonging to space group *P*2_1_2_1_2_1_ with two protein molecules, chains *A* and *B*, in the asymmetric unit. The structure of the enzyme could be solved by molecular replacement using the structure of *H. jecorina* Cel7A (PDB entry 1cel) as the search model, and was refined at 1.8 Å resolution to a final *R*_work_ and *R*_free_ of 0.167 and 0.210, respectively. Details and statistics of data collection and structure refinement are summarized in Table 2 ▶. An example of electron density at the contact between loop B2 and loop A3 in chain *A* is shown in Fig. 4(*b*).

The two noncrystallographically related protein molecules in the asymmetric unit are practically identical along the β-sandwich core of the structure, but deviate at extended loops that enclose the active site, probably owing to different crystal packing. Chains *A* and *B* exhibit 0.62 Å root-mean-square deviation (r.m.s.d.) over 416 C^α^ positions. An overlay of the two chains is shown in Supplementary Fig. S2. The cellulose-binding path is more open in chain *B* than in chain *A*, which will be discussed further below. In chain *A*, amino-acid residues 1–437 could be fitted into electron density. However, two residues at the C-terminus (438–439) were not visible and are not present in the final model of chain *A*. One loop that folds back onto the globular domain in chain *A* to enclose the tunnel at subsites −3/−4 (hereafter called loop B2), appears to be open in chain *B* and is partly disordered. Consequently, eight residues (193–200) at the tip of the loop are omitted in chain *B* of the final structure model owing to insufficient density. On the other hand, the last two residues of the catalytic domain, Pro438 and Gly439, show clear density and are included in chain *B*. The N-terminal glutamine residue is cyclized to pyroglutamate (PCA1) in both chains, and all 18 cysteines form disulfide bonds. *N*-Glycosylation is evident at Asn271 in chain *A*, with density for one *N*-acetylglucosamine residue (NAG), but the density is not clear enough to place an NAG at the corresponding position in chain *B*.

### 3.6. Overall structure of *Hgt*Cel7A and comparison with other GH7 cellobiohydrolases   

**Figure 3:**
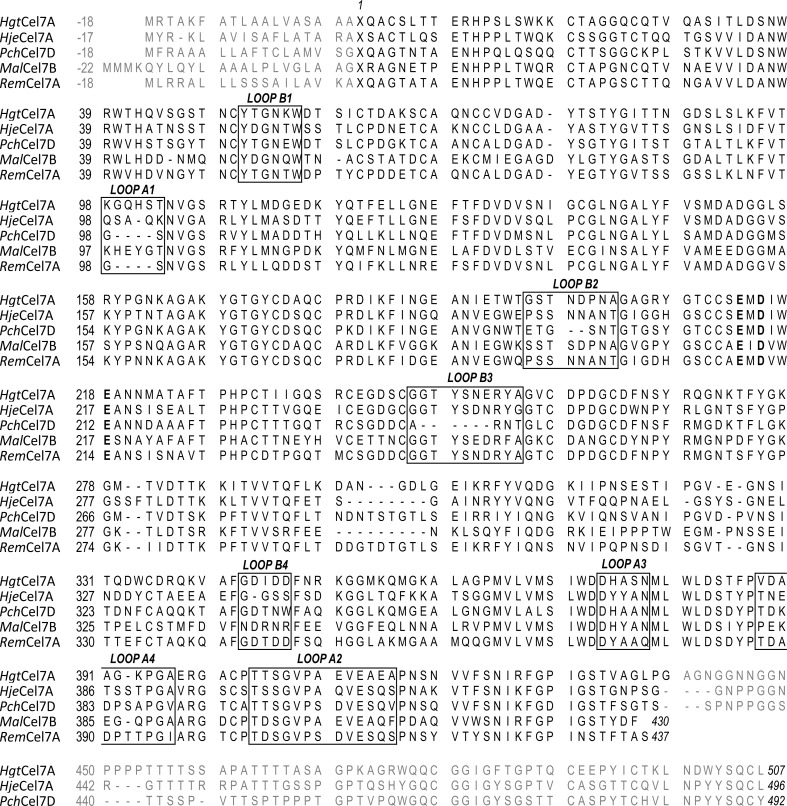
Structure-based sequence alignment of the full-length *Hgt*Cel7A, *Hje*Cel7A (GenBank CAH10320), *Pch*Cel7D (GenBank AAA19802), *Mal*Cel7B (GenBank CAD56667) and *Rem*Cel7A (GenBank AAL89553). The catalytic residues, two glutamates and an aspartate, are highlighted in bold. Loops of interest are indicated by boxes and labelled as in Fig. 4 ▶(*a*).

**Figure 4:**
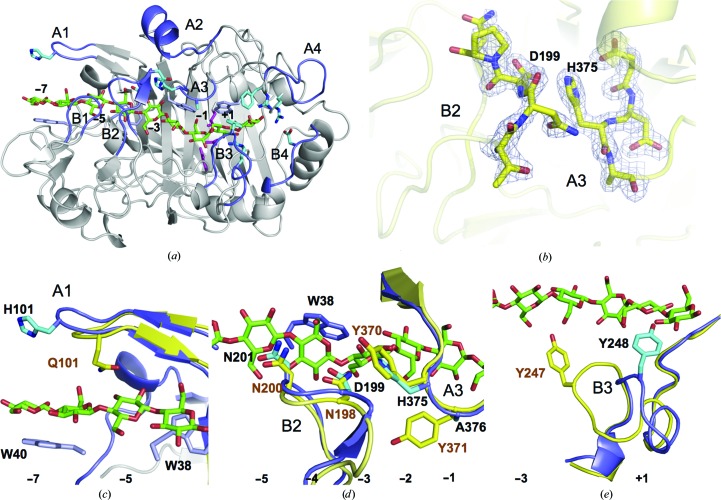
(*a*) Overall structure of *Hgt*Cel7A with a cellulose chain (green) from the *Hje*Cel7A structure (PDB entry 4c4c; Knott *et al.*, 2014 ▶) superimposed. Loops of interest are coloured blue and labelled as in Fig. 3 ▶. Numbers indicate glucosyl-binding subsites. Catalytic residues are shown in magenta, sugar-binding tryptophan platforms in blue-violet and other residues of interest in cyan. In all panels the *A *chain of the *Hgt*Cel7A structure is shown. (*b*) Electron-density map around the tips of loops B2 and A3 contoured at 0.45 e^−^ Å^−3^. (*c*) Superposition of loop A1 at the tunnel entrance of *Hgt*Cel7A (blue) and *Hje*Cel7A (yellow). The *Hgt*Cel7A loop A1 contains a histidine residue (His101) at the tip, and the loop is one residue longer than the corresponding loop in *Hje*Cel7A. (*d*) Superposition of loops A3 and B2 over subsite −4. *Hgt*Cel7A contains His375 and Ala376 instead of Tyr370 and Tyr371, respectively, at the tip of loop A3. (*e*) Loop B3 of *Hgt*Cel7A adopts a new conformation where Tyr248 at the tip is pointing into subsite +2. In *Hje*Cel7A the corresponding Tyr247 instead points towards the −1 subsite.

As expected from the high amino-acid sequence similarity (Fig. 3 ▶), the overall fold of the catalytic domain of *Hgt*Cel7A (Fig. 4 ▶ *a*) is similar to other GH7 CBHs. The r.m.s.d. over all C^α^ positions is 1.0–1.2 Å upon pairwise comparison of *Hgt*Cel7A chain *A* with *Hje*Cel7A (60% sequence identity; PDB entry 8cel; Divne *et al.*, 1998 ▶), *Pch*Cel7D (65%; 1z3v; Ubhayasekera *et al.*, 2005 ▶), *Mal*Cel7B (56%; 2rfw; Parkkinen *et al.*, 2008 ▶) and *Rem*Cel7A (63%; 1q9h; Grassick *et al.*, 2004 ▶). Superposition of *Hgt*Cel7A and *Hje*Cel7A with a model with a cellulose chain bound (PDB entry 8cel; Divne *et al.*, 1998 ▶) demonstrates that the cellulose-binding path is highly conserved, including the catalytic triad Glu213 (nucleophile), Asp215 and Glu218 (acid/base) (residues 212, 214 and 217 in *Hje*Cel7A) and the tryptophan platforms at subsites −7, −4, −2 and +1 (Trp40, Trp38, Trp372 and Trp381 in *Hgt*Cel7A). Nearly all amino acids identified by Divne *et al.* (1998 ▶) as being important for cellulose binding are conserved at similar positions. Major differences that are potentially related to the function of the enzyme are observed at four regions along the substrate-binding path: the tunnel entrance at subsites −7/−6 (loop A1; Fig. 4 ▶ *c*), the loop contacts around subsite −4 (loop B2; Fig. 4 ▶ *d*), near the catalytic centre (loop B3; Fig. 4 ▶ *e*) and adjacent to the product-binding subsites, which are discussed in turn below.

#### 3.6.1. Comparison of the tunnel entrance at subsites −7/−6   

At the entrance to the tunnel the cellulose chain is covered by loop A1, also called the ‘entrance loop’, which varies in both length and sequence among GH7 CBHs. Recent MD simulations of loop dynamics in *Hir*Cel7A (*Heterobasidion irregulare*; Momeni *et al.*, 2013 ▶) and *Lqu*Cel7B (*Limnoria quadripunctata*; Kern *et al.*, 2013 ▶) indicate a potential role in cellulose chain acquisition of a tyrosine residue that is exposed at the tip of loop A1 in both of these enzymes as well as in *Mal*Cel7B (Parkkinen *et al.*, 2008 ▶) owing to interactions with the glucosyl unit at subsite −7. In *Hgt*Cel7A, there is a histidine, His101, instead of tyrosine at the tip of loop A1. His101 may have a similar function, although it is more distant from the −7 glucosyl of the 8cel model compared with the tyrosine in *Mal*Cel7B and *Hir*Cel7A (Figs. 4 ▶ *a* and 4 ▶ *c*). The A1 loop appears to be flexible as observed in other GH7 CBHs since it is shifted outwards in chain *B* compared with chain *A*. Furthermore, the conformation of the loop is likely to be influenced by crystal packing. In both chains *A* and *B* the A1 loop sticks into the tunnel and occupies the −7 subsite of the other protein molecule in the asymmetric unit. Interestingly, the *Hgt*Cel7A sequence BAA09785.1 in GenBank has tyrosine instead of histidine at this position. Loop A1 is shorter by one residue in *Hje*Cel7A and by four residues in *Pch*Cel7D, *Rem*Cel7A and *Tha*Cel7A. All four of these enzymes lack a tyrosine or histidine at the corresponding position.

#### 3.6.2. Comparison of loop contacts near the −4 subsite   

**Figure 5:**
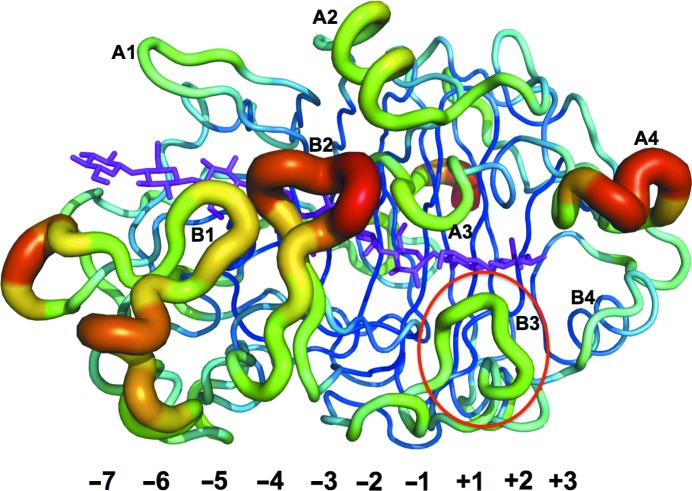
Overall secondary structure of *Hgt*Cel7A (chain *A*) shown in the *B*-factor putty representation of the *PyMOL* program, ramp-coloured from blue to red from low to high temperature factors. The cellononaose chain is taken from the *Hje*Cel7A structure 4c4c (Knott *et al.*, 2014 ▶) superimposed on the *Hgt*Cel7A structure. Loops are labelled as in Figs. 3 ▶ and 4 ▶ and loop B3 is encircled in red. Numbers refer to the glucosyl-binding subsites.

Loop B2 constitutes a 13–15-residue insertion in CBHs relative to GH7 EGs and folds over the β-sandwich core to define the roof of the tunnel around subsite −4. The loop is closed in *Hgt*Cel7A chain *A*, where Asp199 at the tip of the loop interacts with the side chain of His375 on the opposing loop A3 across the tunnel, in analogy with the interaction between the corresponding residues in *Hje*Cel7A: Asn198 and Tyr370 (Fig. 4 ▶ *d*). However, loop B2 appears to be more flexible in *Hgt*Cel7A. In chain *B*, the loop is open and partially disordered, with insufficient density to build residues 193–200, probably owing to interference by crystal contacts with a neighbouring protein molecule that prevents closure of the loop. A similar disorder, presumably owing to loop opening, was observed in the apo structure of *Rem*Cel7A (PDB entry 1q9h; Grassick *et al.*, 2004 ▶) and in *Hir*Cel7A chain *B* (PDB entry 2yg1; Momeni *et al.*, 2013 ▶). Flexibility in loop B2 is further corroborated by the fact that it exhibits the highest temperature factors for main-chain atoms, also in chain *A* of the *Hgt*Cel7A structure where the loop is closed (Fig. 5 ▶). Most GH7 CBH sequences have the same loop B2 length, but the residue on the opposing loop A3 varies, with either His or Tyr being the most common. In *Pch*Cel7D the B2 loop is two residues shorter and does not reach for direct contact across the tunnel.

#### 3.6.3. Comparison of the loops near the catalytic centre   

**Figure 6:**
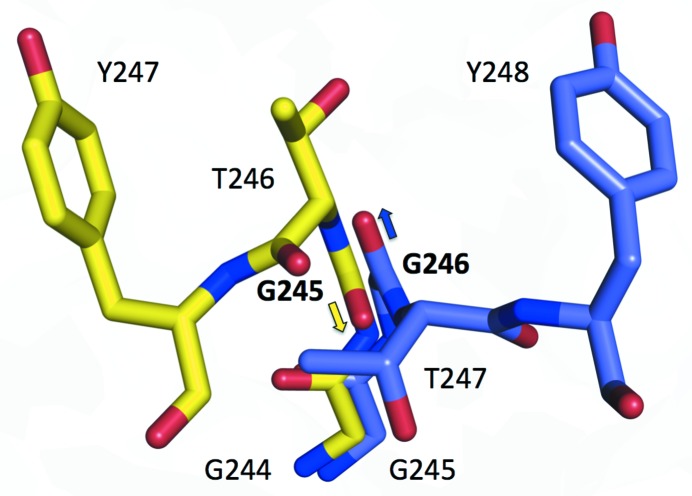
Superposition of the loop B3 hinge in *Hgt*Cel7A (chain *A*, blue) and *Hje*Cel7A (yellow; PDB entry 4c4c; Knott *et al.*, 2014 ▶). Gly246 in *Hgt*Cel7A is rotated almost 180° about the ψ angle compared with Gly245 in *Hje*Cel7A as indicated by the arrows.

Loop B3, residues 245–253 in *Hgt*Cel7A, is also referred to as the exo-loop (von Ossowski *et al.*, 2003 ▶). It has the same length and a similar sequence as in *Hje*Cel7A, *Mal*Cel7B and *Rem*Cel7A, but adopts a different conformation in the *Hgt*Cel7A structure that has not been observed previously in GH7 structures (Fig. 4 ▶ *e*). In *Hje*Cel7A the loop bends towards the catalytic centre; at the tip of the loop Thr246 binds to the substrate at subsite +1 and Tyr247 interacts with both the substrate in subsite −2 and *via* van der Waals contacts with Tyr371 on loop A3 across the tunnel. *Hgt*Cel7A is lacking similar interaction opportunities across the active site, since Tyr371 of *Hje*Cel7A is replaced by Ala376 in *Hgt*Cel7A. In both chains *A* and *B* of the *Hgt*Cel7A structure, loop B3 is instead shifted towards the product-binding sites, where Tyr248 at the tip of the loop points into subsite +2 at a contact distance of about 3.5 Å from Phe386 across the tunnel (corresponding to Tyr381 in *Hje*Cel7A; Fig. 4 ▶ *e*). The shift is accomplished by rotation about the ψ angle of Gly246 by 178° and 162° for chains *A* and *B*, respectively, relative to Gly245 in *Hje*Cel7A. The glycine residue thus acts as a hinge that makes the peptide chain proceed in the opposite direction (Fig. 6 ▶). The largest distance from the corresponding atom in *Hje*Cel7A is shown by the hydroxyl O atom of Tyr248: 11.8 and 12.5 Å for chains *A* and *B*, respectively. Towards the end, loop B3 of *Hgt*Cel7A is in register again with the other structures at the conserved Arg252, which plays a role in substrate interaction at both subsites +1 and +2.

The conformation of loop B3 is similar in chains *A* and *B* of the *Hgt*Cel7A structure, but the loop is shifted closer towards the product sites in chain *B* and Tyr248 penetrates about 1.1 Å deeper into subsite +2. This is probably owing to differences in crystal packing. In chain *B* the loop is covered by a large crystal contact interface and cannot adopt the conformation observed in the structures of the homologous enzymes, since the space is partially occupied by a neighbouring protein molecule. However, in chain *A* there appears to be ample space to switch between these conformations, although the crystal contacts at the periphery of the loop (Asn250 and Glu251) may give some preference to the observed conformation.

It is noteworthy that in the crystal structure Tyr248 at the tip of loop B3 partially obstructs the +2 subsite in both the *A* and the *B* chain. The loop is not likely to adopt these conformations during enzyme action on cellulose. At least, the Tyr248 side chain needs to retract some 1–2 Å from subsite +2.

#### 3.6.4. Comparison of the product-binding region   

The product-binding region of *Hgt*Cel7A is highly conserved in GH7 CBHs. Two important differences in *Hgt*Cel7A are the conformational change of loop B3 mentioned above and the presence of Phe386 in loop A4 near the +2 subsite where there is a tyrosine residue in other GH7 CBH structures and in most of the GH7 CBH sequences (Fig. 4 ▶ *d*). The end of the active-site cleft, beyond the reducing end of the cellulose chain, is defined by loop B4, which exhibits a similar sequence and structure as in other GH7 CBHs. The side chain of Asp344 in loop B4 points towards and can hydrogen bond to the reducing end of the cellulose chain at subsite +2. An aspartate is conserved here in most GH7 CBH sequences, but is missing in *Hypocrea*/*Trichoderma* species owing to a one-residue deletion in loop B4.

## 4. Discussion   

The structure of *Hgt*Cel7A indicates that the loops that surround and define the cellulose-binding path through the enzyme have higher flexibility and mobility relative to those of *Hje*Cel7A. Loops B2 and B3 are of particular interest since they may interact with the opposing loop (A3) across the active site and thereby effectively enclose the active site in a tunnel. A closed tunnel suggests that a cellulose chain may only reach the catalytic centre by threading from the tunnel entrance. However, endolytic cleavage has been experimentally shown for GH7 CBHs, demonstrating that these loops may open occasionally to allow the enzyme to grab an internal part of a cellulose chain (Ståhlberg *et al.*, 1993 ▶; Kurasin & Väljamäe, 2011 ▶). The mobility of tunnel-enclosing loops will obviously dictate the probability of endo-initiation of cellulose hydrolysis. Furthermore, higher flexibility and a more open active site may enhance the rate of enzyme detachment from the cellulose substrate and may also reduce product inhibition, but with a decrease in the degree of processivity as a trade-off (Kurasin & Väljamäe, 2011 ▶; Gruno *et al.*, 2004 ▶; Fox *et al.*, 2012 ▶; Momeni *et al.*, 2013 ▶). Enzyme detachment from the cellulose chain when blocked has been proposed as a key rate-limiting factor for GH7 CBHs (Igarashi *et al.*, 2011 ▶; Jalak & Väljamäe, 2010 ▶; Cruys-Bagger *et al.*, 2012 ▶). Indeed, there seems to be a general trend that a more open active site and/or higher flexibility give faster degradation, at least when the GH7 CBH acts alone on a pure cellulose substrate (von Ossowski *et al.*, 2003 ▶; Kurasin & Väljamäe, 2011 ▶). This is consistent with our results. The high activity of *Hgt*Cel7A on PASC may be owing to the increase in the mobility of the loops that define its active site relative to *Hje*Cel7A.

Loops B2 and B3 of *Hgt*Cel7A have the same length and a similar sequence as in *Hje*Cel7A and also have very similar surroundings. This suggests that the reasons for the difference in behaviour may not reside within the loops themselves. Rather, we believe that the dynamics of these loops are primarily governed by their interaction opportunities across the active site. In particular, two residues at the tip of loop A3 appear to play an important role here. In *Hje*Cel7A, tyrosines 370 and 371 of loop A3 interact with the tips of loops B2 (Asn198) and B3 (Tyr247), respectively. The corresponding residues in *Hgt*Cel7A are His375 and Ala376. His375 is in contact with loop B2 (Asp199) in chain *A*, but not in chain *B*, where loop B2 appears to be open. A histidine is also found in the same position in *Hir*Cel7A, where MD simulations show larger fluctuations in loop B2 and more frequent tunnel opening relative to *Hje*Cel7A, primarily because of a stable hydrogen bond to Tyr370 in the latter enzyme (Momeni *et al.*, 2013 ▶). MD simulations of *T. harzianum* Cel7A (*Tha*Cel7A) and *Hje*Cel7A also point to the importance of loop A3 for the mobility of loop B3 (Textor *et al.*, 2013 ▶). These fungi are closely related and the enzymes share over 80% sequence identity. Loop B3 is nearly identical in these two enzymes, but Tyr371 in loop A3 of *Hje*Cel7A is replaced by an alanine in *Tha*Cel7A (as in *Hgt*Cel7A). In *Hje*Cel7A the loops remain in contact throughout the MD simulation, whereas in *Tha*Cel7A loop B3 shows larger fluctuations and is frequently opened for complete exposure of the active site.

The B3 loop of *Hgt*Cel7A exhibits somewhat elevated *B* factors, although considerably lower than loop B2 (Fig. 5 ▶). Loop B3 adopts a new conformation where Tyr248 points into subsite +2 of the active site, which has not been observed previously in any GH7 structure. For simplicity, we call this the ‘+2 position’ to distinguish it from the predominant ‘−1 position’ observed in other Cel7 homologues, where the tip of the loop points towards the catalytic centre. At this stage we cannot exclude that the ‘+2 position’ observed in *Hgt*Cel7A could be an artefact caused by the crystal packing. In chain *B* the loop is physically hindered by a neighbouring protein from adopting the ‘−1 position’, but not in chain *A*, as explained above. We modelled the B3 loop of *Hgt*Cel7A onto that of *Hje*Cel7A, *i.e.* in the ‘−1 position’, and it seems to fit well into the *Hgt*Cel7A structure without any steric hindrance. This and the fact that the ‘+2 position’ obstructs the +2 subsite and thus appears to be incompatible with enzyme action on cellulose make us believe that loop B3 is flexible and can switch between these two positions in *Hgt*Cel7A. The ‘+2 position’ is apparently preferred in the crystal, but the preference may shift when the enzyme is engaged in cellulose hydrolysis.

Furthermore, we note that in all GH7 structures with this type of B3 loop the loop shows a characteristic conservation pattern and the surroundings are practically identical. The loop is tightly anchored by disulfide bonds at both ends and there are conserved glycines near both ends that may act as hinge points for conformational changes. Superposition of the structures indicates that loop B3 may be able to adopt the ‘+2 position’ in other Cel7 homologues, including *Hje*Cel7A, *Mal*Cel7B and *Rem*Cel7A. Thus, our *Hgt*Cel7A structure points to a new alternate conformation of loop B3 and a putative conformational switch within homologous GH7 CBHs. However, further studies are needed to investigate how often such conformational changes may occur in different enzymes and to elucidate possible connections with enzyme action.

As shown in Fig. 5 ▶, there are several loops with elevated *B* factors near the tunnel entrance, including loops A1, B1 and B2, indicating considerable flexibility in this region. GH7 CBHs operate at the solid–liquid interface, where this region is more or less in contact with the cellulose surface, which is likely to affect the dynamics of the loops as indicated by previous computational studies (Payne *et al.*, 2013 ▶). High *B* factors are also evident for loop A4 adjacent to subsite +2, which may have implications for product expulsion and product inhibition.

Despite its apparently higher flexibility, *Hgt*Cel7A is about 10°C more thermostable than *Hje*Cel7A. The structure of the enzyme thus allows considerable mobility of the surface loops, while avoiding propagation of this movement into the core of the protein structure that could lead to irreversible protein unfolding. Upon closer examination of the base of certain loops, *i.e.* the regions where they connect to the secondary-structure framework, some potentially stabilizing interactions were recognized.

Gln43 and Ile60 at the base of loop B1 in *Hgt*Cel7A make a larger hydrophobic interaction interface than the corresponding residues in *Hje*Cel7A (Ala and Leu, respectively). This may have a stabilizing effect primarily on the 43–48 region, which appears to be rather loosely connected at the surface of the protein near the tunnel entrance. In *Mal*Cel7B the corresponding Asp and Ala residues are not in contact with each other. In *Rem*Cel7A the residues are replaced by Asp and Tyr, but the Asp side chain exhibits elevated temperature factors, indicating substantial fluctuations here.

The long and remarkably mobile loop B2 is anchored by a salt bridge between Glu191 and Arg206 at the N- and C-termini of the loop (Supplementary Fig. S3). The glutamine is conserved in most of the structures, but an arginine at this position is unique to *Hgt*Cel7A. Arg206 is also involved in a salt bridge with Asp240 at the base of loop B3, cross-linking these regions, and may have a crucial stabilizing role in *Hgt*Cel7A.

The mobility of loop A4 (387–396) is restricted by conserved proline residues at both ends. At the N-terminal side the proline is preceded by Phe386 in *Hgt*Cel7A or a tyrosine in most other GH7 CBHs, which is well embedded and holds the loop in place. At the C-terminal side of loop A4, Glu397 makes an additional hydrogen bond (to Tyr267) that is not present in the other Cel7 structures because the glutamate is substituted by alanine (except in *Hje*Cel7A, which has a valine at this position).

At the C-terminus of the catalytic domain the side chains of Val434 and Leu437 (glycine and serine in *Hje*Cel7A) form a hydrophobic cluster together with Val290, Phe307 and Ile314. This indicates that the linker peptide is more firmly anchored and that the native full-length *Hgt*Cel7A may tolerate larger dynamics of the linker–CBM tail without propagation of unfolding into the core of the catalytic domain.

Finally, the Cel7A cellobiohydrolase from *H. grisea* var. *thermoidea* was successfully expressed in both *A. awamori* and *H. jecorina* and was shown to be considerably more thermostable than *Hje*Cel7A, with a 10°C higher *T*_m_. The crystal structure of the enzyme reveals considerable flexibility of the active-site-defining loop regions and an alternate conformation of loop B3 that has not been observed previously in GH7. The *Hgt*Cel7A exhibits much higher activity than *Hje*Cel7A when assayed alone on PASC as substrate, most likely owing to the higher loop mobility. In a performance assay at elevated temperature (65°C) on PCS, together with a *H. jecorina* enzyme cocktail, the enzyme gave about a 75% higher yield of soluble sugar than *Hje*Cel7A. Thus, *Hgt*Cel7A is a promising GH7 cellobiohydrolase candidate with potential for exploitation in biomass-conversion applications.

## Supplementary Material

Supporting Information.. DOI: 10.1107/S1399004714013844/rr5073sup1.pdf

PDB reference: Cel7A, 4csi

## Figures and Tables

**Table 1:** Cel7 enzymes expressed in *A. niger* var. *awamori* AP4 and estimated *T*_m_ values

Species	Strain	Sequence†	% identity‡	*T*_m_ (°C)
*Hypocrea jecorina*	ATCC 13631	CAH10320.1	100	62.5
*Hypocrea orientalis*	PPRI 3894	§ ¶	97	62.8
*Hypocrea schweinitzii*	CBS 243.63	§ ¶	96	61.4
*Trichoderma pseudokoningii*	CBS 408.91	§ ¶	95	57.5
*Trichoderma citrinoviride*	DAOM 196.431	ACH96125.1	94	62.6
*Trichoderma konilangbra*	Isolate 1	§ ¶	93	59.4
*Aspergillus niger*	FGSC A237	Q9UVS8¶ ††	58	59.3
*Aspergillus aculeatus*	CBS 610.78	AB002821	57	63.7
*Penicillium janthinellum*	CBS 340.48	X59054	57	63.3
*Humicola grisea* var. *thermoidea*	CBS 225.63	D63515‡‡	56	72.5

† Accession code for the sequence from which primers were developed and to which the sequence of the expressed protein is identical unless indicated otherwise.

‡ Percentage sequence identity with *H. jecorina* Cel7A.

§ Primers for *H. jecorina* Cel7A were used here.

¶ The sequence of the retrieved Cel7 homologue is shown in Goedegebuur *et al.* (2011 ▶).

†† The Cel7 retrieved from *A. niger* showed 18 amino-acid differences from the published Q9UVS8 sequence, indicating that another Cel7 gene was amplified and expressed.

‡‡ The Cel7 retrieved from *H. grisea* var. *thermoidea* shows one amino-acid difference from the published sequence, as described in the text.

**Table 2:** X-ray data-collection, processing and structure-refinement statistics for *Hgt*Cel7A Values in parentheses are for the highest resolution shell.

Data collection	
Resolution range	34.71–1.80 (1.90–1.80)
Wavelength (Å)	0.93
No. of unique reflections	65221 (35670)
Space group	*P*2_1_2_1_2_1_
Unit-cell parameters (Å)	*a* = 59.9, *b* = 85.3, *c* = 135.8
Completeness (%)	99.8 (99.8)
Multiplicity	3.9 (3.8)
*R*_merge_ † (%)	8.6 (41.0)
Mean *I*/σ(*I*)	7.1 (1.9)
Refinement	
*R*_work_/*R*_free_ (%)	16/21 (25/32)
R.m.s.d., bond lengths (Å)	0.009
R.m.s.d., bond angles (°)	1.3
Wilson *B* factor (Å^2^)	17.6
No. of atoms	
Protein	6630
Carbohydrate	42
Water molecules	718
Mean *B* factors (Å^2^)	
Protein (chain *A*/*B*)	16.53/17.13
Carbohydrate	25.31
Water	24.7
Ramachandran plot‡, residues in (%)	
Favoured region	95.5
Allowed region	0.5
PDB entry	4csi

† *R*_merge_ = 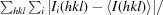 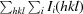.

‡ Calculated using a strict-boundary Ramachandran plot (Kleywegt & Jones, 1996 ▶)

## Footnotes

1 Supporting information has been deposited in the IUCr electronic archive (Reference: RR5073).
