# Supplementary material for: Expression, crystal structure and cellulase activity of the thermostable cellobiohydrolase Cel7A from the fungus Humicola grisea var. thermoidea
Source: Acta Crystallogr D Biol Crystallogr. 2014 Aug 29;70(Pt 9):2356–66. doi: 10.1107/S1399004714013844 (PMC4157447; doi:10.1107/S1399004714013844)
Supplement: Supplementary file 1 [file d-70-02356-sup1.pdf]

Acta Crystallographica Section D

Volume 70 (2014)

Supporting information for article:

Expression, crystal structure and cellulase activity of the thermostable Cellobiohydrolase Cel7A from the fungus *Humicola grisea* var. *thermoidea*

Majid Haddad Momeni, Frits Goedegebuur, Henrik Hansson, Saeid Karkehabadi, Glareh Askarieh, Colin Mitchinson, Edmundo A. Larenas, Jerry Ståhlberg and Mats Sandgren

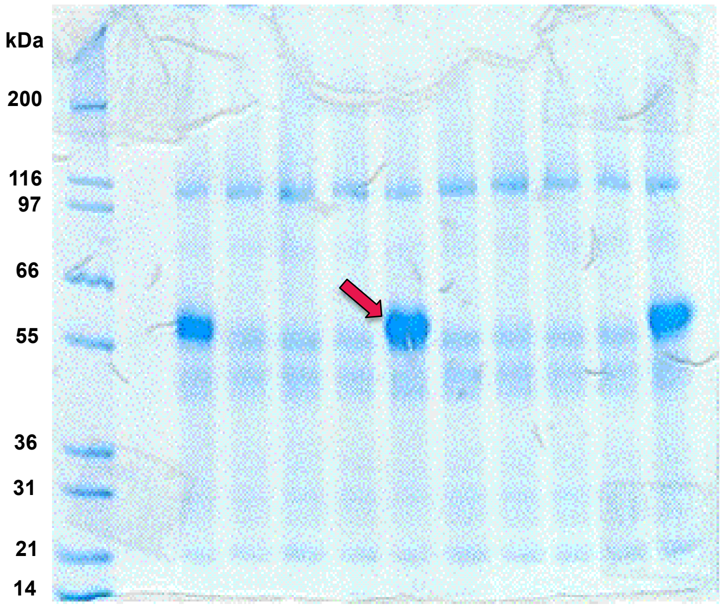

Figure S1 SDS-PAGE gel. The arrow indicates the *H. grisea* var. *thermoidea* Cel7A protein band in culture filtrate from expression of the enzyme in a *Hypocrea jecorina* strain deleted for the four major cellulases (*cbh1*$^-$, *cbh2*$^-$, *egl1*$^-$, *egl2*$^-$), as described in the main article.

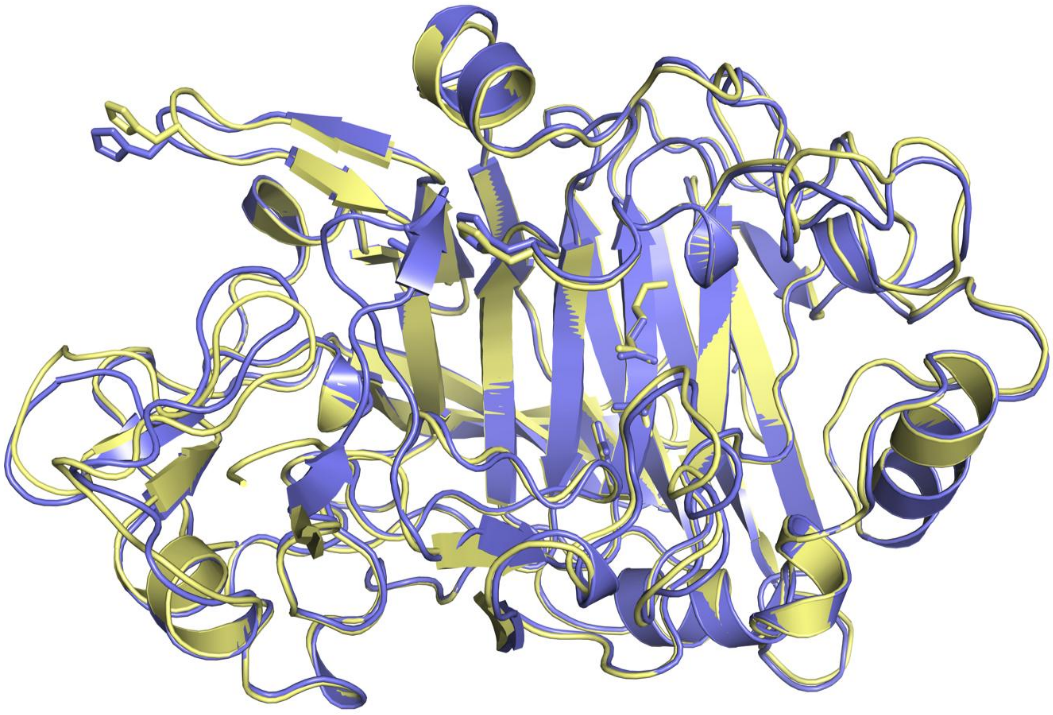

Figure S2 Superposition of the backbones of chain A (blue) and chain B (yellow) of the *Humicola grisea* var. *thermoidea* Cel7A catalytic domain structure (4CSI).

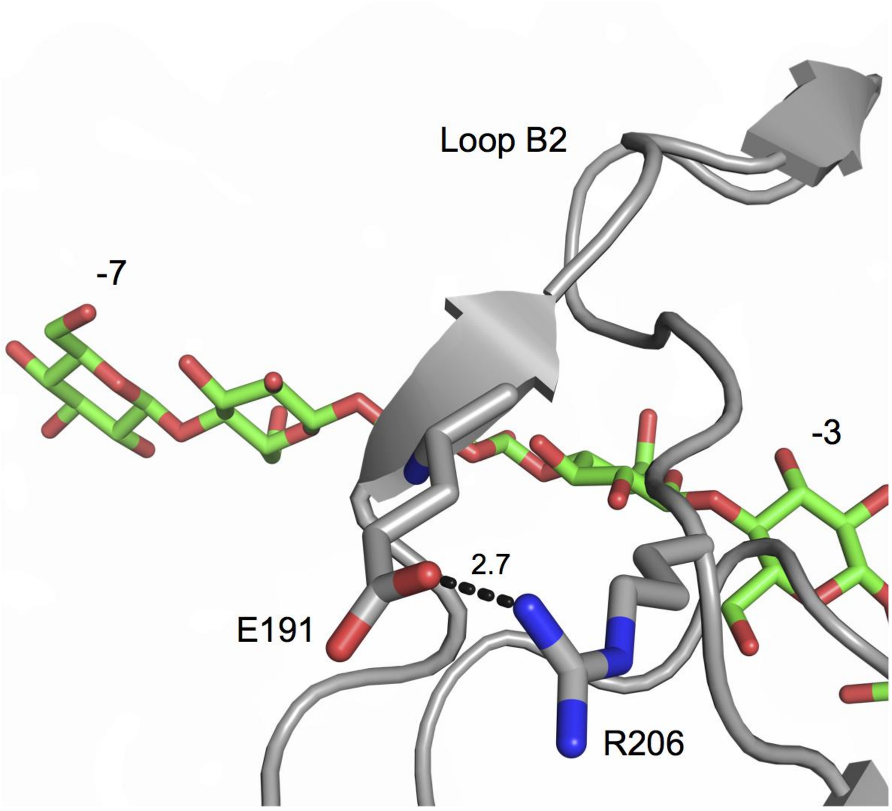

Figure S3 Salt bridge hydrogen bonding between Glu191 and Arg206 stabilizes loop B2 at the base of the loop. The cellodextrin chain (green) is taken from the *H. jecorina* Cel7A Michaelis complex 4C4C. Glucosyl-binding subsites -7 and -3 are indicated.
